# Genome-Wide Association Study to Identify Marker–Trait Associations for Seed Color in Colored Wheat (*Triticum aestivum* L.)

**DOI:** 10.3390/ijms25073600

**Published:** 2024-03-22

**Authors:** Min Jeong Hong, Chan Seop Ko, Dae Yeon Kim

**Affiliations:** 1 Advanced Radiation Technology Institute, Korea Atomic Energy Research Institute, 29 Geumgu, Jeongeup 56212, Republic of Korea; hongmj@kaeri.re.kr (M.J.H.); chansubi@kaeri.re.kr (C.S.K.); 2 Department of Plant Resources, College of Industrial Sciences, Kongju National University, 54 Daehak-ro, Yesan-eup 32439, Republic of Korea

**Keywords:** colored wheat, genome-wide association studies, marker–trait associations, seed development stages, transcription profiling

## Abstract

This study conducted phenotypic evaluations on a wheat F_3_ population derived from 155 F_2_ plants. Traits related to seed color, including chlorophyll a, chlorophyll b, carotenoid, anthocyanin, L*, a*, and b*, were assessed, revealing highly significant correlations among various traits. Genotyping using 81,587 SNP markers resulted in 3969 high-quality markers, revealing a genome-wide distribution with varying densities across chromosomes. A genome-wide association study using fixed and random model circulating probability unification (FarmCPU) and Bayesian-information and linkage-disequilibrium iteratively nested keyway (BLINK) identified 11 significant marker–trait associations (MTAs) associated with L*, a*, and b*, and chromosomal distribution patterns revealed predominant locations on chromosomes 2A, 2B, and 4B. A comprehensive annotation uncovered 69 genes within the genomic vicinity of each MTA, providing potential functional insights. Gene expression analysis during seed development identified greater than 2-fold increases or decreases in expression in colored wheat for 16 of 69 genes. Among these, eight genes, including transcription factors and genes related to flavonoid and ubiquitination pathways, exhibited distinct expression patterns during seed development, providing further approaches for exploring seed coloration. This comprehensive exploration expands our understanding of the genetic basis of seed color and paves the way for informed discussions on the molecular intricacies contributing to this phenotypic trait.

## 1. Introduction

Since its domestication approximately 10,000 years ago, wheat (*Triticum aestivum* L.) has become a cornerstone of global food security, contributing significantly to meeting the dietary needs of the global population. Its widespread cultivation and consumption have established wheat as a primary source of calories and protein, providing sustenance for a substantial portion of the global population [1]. This unique variation in wheat both adds to its nutritional profile and holds promise for enhancing the overall dietary diversity and health benefits available to consumers [2]. Wheat provides important nutrients and compounds such as anthocyanins, carotenes, and phenolic acids, which have strong antioxidant effects [3]. Colored wheat, with its anthocyanin content, has a powerful ability to combat chronic diseases such as obesity, cancer, and cardiovascular issues, and it can even slow aging [4]. In contrast to common wheat, the red color of which arises from carotenoids and catechol in the outer layer, the color of colored wheat is mainly attributable to anthocyanins. Colored wheat also contains many tocopherols, phenolic acids, and essential trace elements needed for the human body [5,6].

The transformative impact of single nucleotide polymorphism genotyping arrays (SNP arrays) extends beyond their pivotal role in exploring genetic variations in both animal and plant populations [7,8]. By facilitating the identification and analysis of hundreds of thousands of SNPs in a single assay, these arrays serve as a robust platform for unveiling genome-wide sequence variability among individuals and populations [9]. SNP arrays provide a high-throughput and cost-effective method for analyzing genetic diversity, and they have been extensively employed in constructing genetic linkage maps, exploring evolutionary relationships, unraveling functional genomics, and supporting conservation efforts. Genotyping arrays have played, and continue to play, a critical role in the genotyping of various crop species. Consequently, the common study of SNPs often identifies loci that are blocks of correlated SNPs associated with the trait of interest [10].

In recent decades, high-density SNP genotyping arrays such as Illumina Wheat 9K, 90K, 15K, Axiom^®^ Wheat 660K, Wheat 55K, Axiom^®^ HD Wheat (820K), Wheat Breeders’ 35K Axiom, and Wheat 50K Triticum TraitBreed arrays have been developed for marker-assisted breeding in common wheat [11,12,13,14,15,16]. This technology facilitates the rapid genotyping of wheat varieties, precise identification of genetic variants linked to crucial traits, and marker development for easy integration into breeding programs. High-density genotyping arrays significantly increase researchers’ ability to study many wheat samples, making it easier to identify genetic variations and advanced wheat breeding techniques.

In this study, we used the F_3_ population of both colored and noncolored wheat lines to identify loci associated with seed color using the comprehensive Illumina Wheat 90 K SNP array. In addition, we explored the mechanisms governing changes in seed color through a comparative analysis of RNA sequences during the seed developmental stages of colored and noncolored wheat. By integrating the results of genome-wide association studies (GWASs) and RNA sequencing (RNA-Seq), we unraveled the changes in expression in differentially expressed genes (DEGs) located near quantitative trait loci that regulate seed color. This collaborative approach sought to enhance our understanding of the complex mechanisms governing seed coloration in wheat, with GWASs providing valuable insights into genetic associations, complemented by a detailed exploration of gene expression patterns through RNA-Seq. In addition, the findings from this study offer novel insights into potential candidate genes influencing wheat seed coloration, particularly during the critical seed filling and maturity stages.

## 2. Results

### 2.1. Phenotypic Evaluations

**Figure 1:**
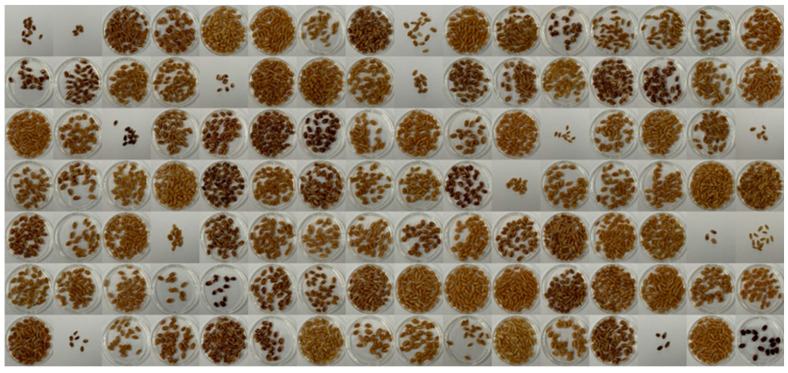
F_3_ population seed images. The figure displays seed images representative of the F_3_ population used in this experiment showing the observed variation in seed color. The F_3_ population originated from a crossbreeding of recombinant inbred lines (RILs) with distinct seed coat phenotypes, namely, yellow (accession no. 10DS1673, sourced from the Korea University Wheat Subgene bank) and deep purple (accession no. 10DS1674). The intentional inclusion of RILs with diverse seed coat phenotypes contributed to the generation of a genetically heterogeneous population, facilitating the exploration of seed color-related traits in subsequent analyses.

**Figure 2:**
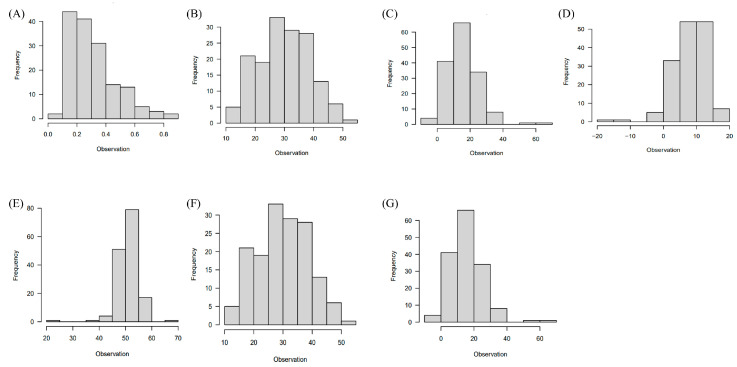
Phenotypic trait distribution. (**A**–**G**) Histograms illustrating the distribution of phenotypic traits in the F_3_ population, including (**A**) anthocyanin, (**B**) chlorophyll a, (**C**) chlorophyll b, (**D**) carotenoid, (**E**) L*, (**F**) a*, and (**G**) b*.

Images of the F_3_ seeds are presented in Figure 1. Of the initial 214 individuals in the F_3_ segregated population, some seeds, including damaged or broken ones, were excluded, resulting in 155 F_3_ plants available for this study. This subset of 155 F_3_ plants was evaluated for traits related to seed color, encompassing chlorophyll a, chlorophyll b, carotenoid, anthocyanin, L*, a*, and b*. The distribution of the results from the phenotype evaluation is depicted in Figure 2A–G, and essential summary statistics, including range, mean, and coefficient of variation, are presented in Table S2.

Pearson’s correlation coefficient (r) estimated between the traits in the F_3_ population is presented in Figure S1. The associations were positive and highly significant (all *p* < 0.01) among carotenoid, chlorophyll a, and anthocyanin; L* and carotenoid; a* and L*; b* and carotenoid; and L* and a*. By contrast, strong negative correlations were detected between carotenoid and chlorophyll b, L* and anthocyanin, and b* and anthocyanin (all *p* < 0.01) in the F_3_ population (Figure S1).

### 2.2. Phenotypic Evaluation of Marker Distribution, Population Structure, and Linkage-Disequilibrium (LD) Decay

Of the 81,587 SNP markers initially present on the wheat 90K iSelect array for genotyping, 3969 high-quality SNP markers remained after eliminating those with minor allele frequencies <0.05 and missing data >10%. The selected SNP markers exhibited a genome-wide distribution, with the highest number on the A subgenome (2500), followed by the B (1249) and D (218) subgenomes. An analysis of their chromosome-wide distributions revealed the highest marker density on chromosome 2A (653), followed by chromosomes 1A (591) and 2B (315). Conversely, chromosomes 5D (11) and 7D (20) contained the fewest markers (Table S3).

**Figure 3:**
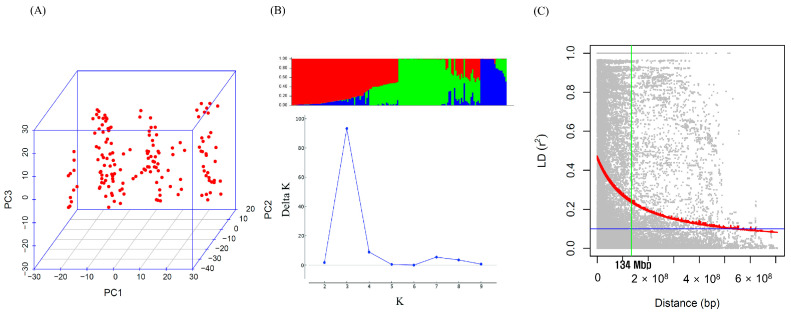
Genotype analysis and linkage-disequilibrium (LD) decay. (**A**) Principal component analysis of 155 genotypes using 3969 single nucleotide polymorphisms provides insights into the genetic relationships among individuals. (**B**) Population structure analysis with three clusters reveals distinct subgroups within the 155 genotypes, enhancing our understanding of the genetic diversity of the population. (**C**) The LD decay plot depicts the genome-wide decay of LD with genetic distance. The region in which LD decays to half is highlighted in green, and 50% decay occurred at 134 Mb across the genome.

The population structure of the 155 wheat genotypes was examined using the ΔK method and validated using principal component analysis (PCA). The ΔK method and PCA-based population structure analysis identified three distinct groups in the GWAS results (Figure 3A,B). LD decay was estimated by calculating r^2^ for all 3969 markers. Genome-wide LD decayed with genetic distance, and LD decayed by 50% at 134 Mb for the entire genome (Figure 3C).

### 2.3. GWASs

**Figure 4:**
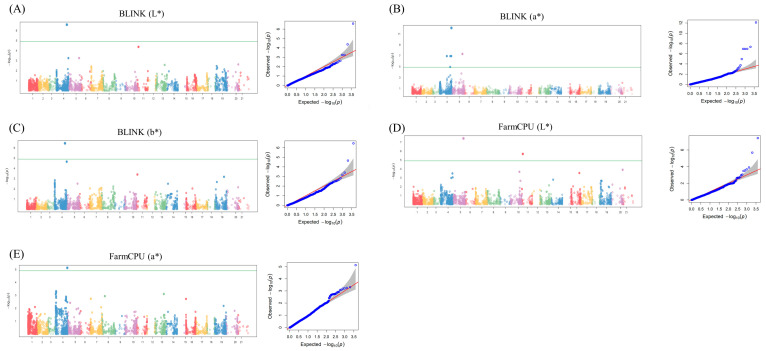
Manhattan and Q–Q plots for significant MTAs. (**A**) Manhattan and Q–Q plots for BLINK (L*) analysis, illustrating genomic regions with significant associations with the L* trait in wheat. (**B**) Manhattan and Q–Q plots for BLINK (a*), highlighting significant MTAs related to a* in the wheat genome. (**C**) Manhattan and Q–Q plots for BLINK (b*), revealing genomic loci significantly associated with b* in wheat. (**D**) Manhattan and Q–Q plots for FarmCPU (L*), displaying genomic regions with noteworthy associations with the L* trait using FarmCPU. (**E**) Manhattan and Q–Q plots for FarmCPU (a*), presenting significant MTAs related to a* in wheat through FarmCPU.

The significant MTAs for the seven phenotypic traits were identified by scrutinizing Q–Q and Manhattan plots in GWAS using FarmCPU and BLINK (Figure 4A–E). The application of a stringent threshold (−log_10_P > 5) served as a robust criterion for designating MTAs as significant in the GWAS. The analysis revealed eleven MTAs, including three from FarmCPU and eight from BLINK (Table 1). All 11 MTAs originated from BLINK (L*, a*, and b*) and FarmCPU (L* and a*). Notably, some MTAs were detected by multiple methods, such as BS00067992_51 (detected in FarmCPU L* and BLINK), Ra_c13247_528 (detected in BLINK L* and a*), and RAC875_rep_c105150_1024 (duplicated in FarmCPU a* and BLINK a*). The phenotypic variation explained (PVE) by these SNPs ranged between 0.17% and 86.08%. In BLINK (a*), the SNP with the lowest PVE was Ra_c13247_528 (0.17%). Interestingly, these specific SNPs were also detected by BLINK (L*), albeit with a significantly higher PVE of 19.64%. In addition, the analysis of the chromosomal distribution of MTAs revealed distinct patterns, with the majority being located on chromosomes 2A, 2B, and 4B. Specifically, six MTAs were identified on chromosome 2A, whereas chromosomes 2B and 4B each harbored one MTA (Table 1).

**Figure 5:**
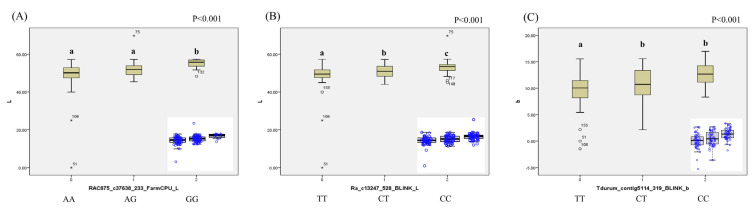
Box plots of allelic differences of significant MTAs. (**A**) Allelic differences for the significant MTAs identified via FarmCPU analysis for L* in wheat. (**B**) Allelic differences for significant MTAs identified via BLINK analysis for L* in wheat. (**C**) Allelic differences for significant MTAs identified via BLINK analysis for b* in wheat. Statistical analysis was performed using ANOVA followed by Duncan’s post hoc analysis (*p* < 0.001) to assess significant differences in mean phenotypic values among genotypes with different allelic variants. Different letters indicate statistically significant differences and the blue circles represent the distribution of lines based on alleic differencesMTA, marker–trait association; BLINK, Bayesian-information and linkage-disequilibrium iteratively nested keyway; FarmCPU, fixed and random model circulating probability unification.

Three SNPs, namely, RAC875_c37638_233, Ra_c13247_528, and Tdurum_contig5114_319, are illustrated in Figure 5, and these SNPs originated from FarmCPU (L*), BLINK (L*), BLINK (a*), and BLINK (b*) (note: BLINK (L*) and BLINK (a*) denote duplicated MTAs). RAC875_c37638_233, positioned on chromosome 4B with a PVE of 6.00% from FarmCPU (L*), exhibited A and G alleles and significantly different mean phenotypic values of L* across genotypes (AA, AG, GG; Figure 4A,D). Meanwhile, Ra_c13247_528 b, located on chromosome 2A with PVEs of 0.18% and 19.94% from BLINK (a*) and BLINK (L*), respectively, displayed C and T alleles with significantly different mean phenotypic values of L* across genotypes (CC, CT, TT). In addition, Tdurum_contig5114_319, situated on chromosome 2A with a PVE of 18.96% from BLINK (b*), displayed significant differences among genotypes (CC, CT, TT).

### 2.4. Gene Expression Analysis during Seed Development in the Vicinity of MTAs

**Figure 6:**
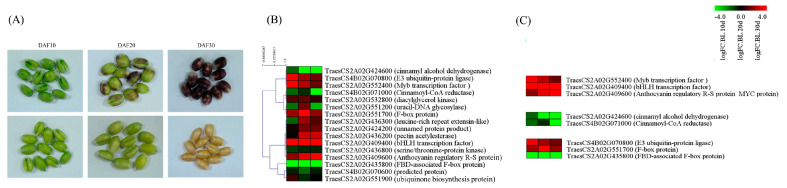
RNA sequencing results and DEGs positioned within a 250 kb radius of the MTAs. (**A**) Images of seed samples during seed developmental stages at 10 DAF, 20 DAF, and 30 DAF used for RNA sequencing. Heatmaps illustrating (**B**) sixteen genes displaying a greater than 2-fold difference in gene expression between colored and non-colored wheat during the seed developmental stage, and (**C**) DEGs involved in transcription factors, phenylpropanoid compounds, and E3 ubiquitin ligase.

**Figure 7:**
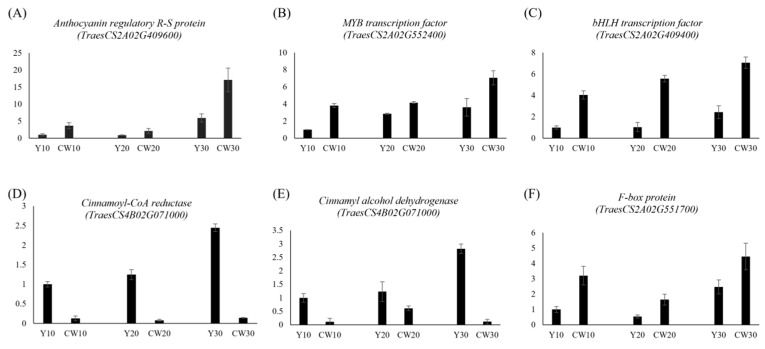
Validation of RNA sequencing data using RT-qPCR analysis. (**A**) Expression patterns of anthocyanin regulatory R-S protein (MYC protein, *TaesCS2A02G409600*), (**B**) MYB transcription factor (*TraesCS2A02G552400*), (**C**) bHLH transcription factor (*TraesCS2A02G409400*), (**D**) cinnamoyl-CoA reductase (*TraesCS4B02G071000*), (**E**) cinnamyl alcohol dehydrogenase (*TraesCS4B02G071000*), and (**F**) F-box protein (*TraesCS2A02G551700*). Biological replicates were used in triplicate, and error bars indicate standard errors.

To gain deeper insights into the genomic context of these MTAs, a comprehensive annotation was conducted using IWGSC Wheat RefSeq v1.1. This annotation effort uncovered a noteworthy discovery. Specifically, 69 genes were identified within the genomic vicinity of each significant MTA locus (Table S4). These genes, positioned within a 250 kb radius of the MTAs, present a rich source for further exploration and potential functional implications related to the observed phenotypic traits. Based on the RNA-Seq data, 16 of 69 genes displayed a greater than 2-fold difference in gene expression between colored and noncolored wheat during seed developmental stages (10 DAF, 20 DAF, and 30 DAF; Figure 6A,B). Two genes (*TraesCS2A02G424200*, and *TraesCS2A02G424600*) were found in close proximity to the MTA associated with L*, whereas five genes (*TraesCS2A02G532800*, *TraesCS2A02G436300*, *TraesCS2A02G436800*, *TraesCS2A02G436200*, and *TraesCS2A02G435800*) were located near the MTA linked to a*. In addition, two genes (*TraesCS2A02G409400* and *TraesCS2A02G409600*) on chromosome 2A were near the MTA related to b*. All these genes were identified via BLINK analysis (Table 2). Two genes on chromosome 4A (*TraesCS4B02G070800* and *TraesCS4B02G071000*) and four genes on chromosome 2A (*TraesCS2A02G551200*, *TraesCS2A02G551900*, *TraesCS2A02G551700*, and *TraesCS2A02G552400*) were found to be closely associated with L* and a*, as identified via FarmCPU analysis. The expression patterns of all 16 genes during the seed developmental stages are illustrated in Figure 6B. Among them, eight genes, categorized as transcription factors, flavonoid pathway-related genes, and ubiquitination pathway genes, were selected, and their expression patterns are depicted in Figure 6C. To assess the reliability of the RNA-Seq results, RT-qPCR was employed to validate the expression profiles of selected genes, including anthocyanin regulatory R-S protein (MYC protein, *TaesCS2A02G409600*), MYB transcription factor (*TraesCS2A02G552400*), bHLH transcription factor (*TraesCS2A02G409400*), cinnamoyl-CoA reductase (CCR, *TraesCS4B02G071000*), cinnamyl alcohol dehydrogenase (CAD, *TraesCS4B02G071000*), and F-box protein (*TraesCS2A02G551700*). The RT-qPCR results were consistent with the RNA-Seq findings, confirming the concordance between the two independent methods. These genes were specifically chosen from the MYB–bHLH–WD40 (MBW) complex, lignin pathway, and E3 ubiquitin ligase categories (Figure 6C) for comprehensive validation, and the congruence of the results further strengthens the robustness of our findings (Figure 7).

## 3. Discussion

In this study, we conducted a comprehensive examination of seven phenotypic traits within an F_3_ population derived from both colored and noncolored wheat using GWASs. The variability in the range of each phenotypic dataset was notable, including coefficients of variation surpassing 50% for chlorophyll b, carotenoid, and anthocyanin contents (59.22%, 51.77%, and 54.84%, respectively). This substantial variation likely influenced the outcomes, as evidenced by all 11 significant MTAs being associated with the phenotypic traits L*, a*, and b*. Considering these results, the observed high interrelation among L*, a*, and b* represents a noteworthy observation. This robust correlation indicates potential associations among these phenotypic traits, suggesting the possibility of shared genetic or biochemical pathways influencing seed color. The CIELAB color space, comprising the L*, a*, and b* channels, captures distinct aspects of color perception, such as lightness, the green–magenta spectrum, and the blue–yellow spectrum. These channels, reflecting specific color attributes, might hold associations with underlying biological factors [17]. In particular, an increase in anthocyanin content was negatively correlated with L* and b*, suggesting that as anthocyanin levels rise, seed brightness decreases, manifesting in a blue–yellow spectrum shifting toward the blue end.

The relationships among the genotypes were analyzed using two distinct methods as follows: subgrouping analysis based on population structure and PCA. Both analyses identified three consistent subgroups, affirming the reliability of the genotype analysis. LD decay over genetic or physical distance in a population influences the marker coverage density required for effective GWASs. More rapid LD decay implies the necessity of higher marker density to capture markers in close proximity to causal loci [17]. In this study, LD decayed to half of its maximum value at 134 Mb across the entire genome. Wheat, being a self-pollinating species with an extremely large genome, exhibits a larger LD decay distance than other plants, including maize [18,19]. Moreover, LD decay can vary among mapping populations of the same species, as observed in Chinese wheat landrace (5.98 Mb) and Mexican bread wheat (22.85 Mb) [20,21]. These variations are likely attributable to differences in cultivation practices, breeding methods, breeding history, and evolutionary history [22]. Additionally, the use of recombinant inbred lines (RILs) with distinct seed coat phenotypes, namely, noncolored wheat (yellow) and colored wheat (deep purple), in the development of the F_3_ population could be one reason for the observed higher LD decay distance.

In this study, BLINK and FarmCPU analyses identified eight MTAs associated with L*, a*, and b* traits. Furthermore, among the 69 genes near these eight MTAs, 16 exhibited significant expression patterns during seed developmental stages, and the corresponding expression patterns of these genes were also determined. Interestingly, the anthocyanin regulatory R-S protein (*TraesCS2A02G409600*), a MYC transcription factor with a basic helix–loop–helix motif, demonstrated continuous upregulation during seed development in colored wheat both in the results of RNA-Seq and RT-qPCR, underpinning its role as a key regulator of anthocyanin structural genes [23]. Moreover, the MYB transcription factor (*TraesCS2A02G552400*) and bHLH transcription factor (*TraesCS2A02G409400*) were also highly expressed during seed developmental stages in colored wheat. MBW protein complexes, which comprise MYB, bHLH, and WD40 repeat factors, are recognized as transcriptional regulators governing the production of secondary metabolites, including proanthocyanidins and anthocyanins [24]. These regulatory elements assemble into the ternary complex MBW, and this complex might utilize alternative MYB and bHLH components to regulate specific steps in the biosynthetic pathways of proanthocyanidins and anthocyanins [25,26].

Phenylpropanoid compounds, including flavonoids and lignin, consist of numerous secondary metabolites that are widely distributed in various tissues and organs of plants. The biosynthesis of lignin and flavonoids shares the early enzymatic steps of the phenylpropanoid pathway before diverging into the flavonoid and lignin pathways [27]. Shi et al. (2022) reported the mechanism underlying the homeostatic regulation of flavonoid and lignin biosynthesis in the phenylpropanoid pathway of plants [28]. In this study, CCR (*TraesCS4B02G071000*) and CAD (*TraesCS2A02G424600*), which are involved in specific steps of the monolignol pathway, were downregulated during seed developmental stages in colored wheat, as demonstrated by both RNA-Seq and RT-qPCR. Moreover, similar trends have been reported in *Arabidopsis* in which mutant lines deficient in CCR and CAD genes accumulate higher amounts of flavonol glycosides in the stem, indicating a redirection of the phenolic pathway [29].

The ubiquitin–proteasome system, which regulates selective protein degradation via the 26S proteasome, is a key mechanism for the post-translational regulation of gene expression and protein quality control in eukaryotes [30]. This system plays a pivotal role in governing signal transduction, metabolic processes, differentiation, cell cycle transitions, and stress responses by orchestrating the degradation of specific proteins [31,32]. Ubiquitin E3 ligases, which are conserved throughout eukaryotes, perform diverse regulatory functions by catalyzing the covalent attachment of ubiquitin to target proteins [33]. The *Arabidopsis* genome encodes more than 1500 E3 ubiquitin ligase proteins, which are categorized into various families such as the HECT, RING1, Kelch-type, U-box, and Cullin–RING ligase (CRL) families. Among these, the F-box protein operates as a component of the SKP1–Cullin–F-box complex within the CRL family of E3 ubiquitin ligases [34,35,36,37,38,39]. Three E3 ubiquitin-protein ligases (*TraesCS4B02G070800*, *TraesCS2A02G551700*, and *TraesCS2A02G435800*), including one RING E3 ubiquitin ligase and two F-box proteins, exhibited significant expression during seed developmental stages. In addition, validation via RT-qPCR analysis revealed that *TraesCS2A02G551700* displayed increased expression in colored wheat during seed developmental stages. Although the specific roles of these E3 ligases in seed coloration remain elusive, further molecular investigations could reveal their functional associations with seed pigmentation. Subsequent research endeavors employing molecular biology approaches could help elucidate the intricate functions linking these E3 ligases to seed coloration.

## 4. Materials and Methods

### 4.1. Plant Materials

RILs with distinct seed coat phenotypes, namely, yellow (accession no. 10DS1673) and deep purple (accession no. 10DS1674) were obtained from Korea University Wheat Subgene Bank [40]. Crossbreeding between yellow and deep purple wheat lines resulted in the generation of F_2_ plants. F_3_ seeds from each of the 155 F_2_ plants were selected for use in this study, with three seeds selected from each plant. Seeds were germinated on moistened filter paper at room temperature for 24 h, followed by vernalization at 4 °C in a dark chamber for 4 weeks. Each seedling was then transferred to a Magenta box (6.5 × 6.5 × 20 cm^3^, Greenpia Technology Inc., Seoul, Republic of Korea) containing polypro mesh. Seedlings were grown in Magenta boxes filled with 180 mL of water for 14 days in the growth facility at 23 °C and a day/night photoperiod of 16 h/8 h.

### 4.2. Anthocyanin and Chlorophyll Content Analysis

For anthocyanin content, homogenized F_3_ wheat seeds were mixed with 1 mL of methanol–hydrochloric acid (1% HCl, *w*/*v*) and incubated at 4 °C for 24 h. The absorbance was measured at 530 and 657 nm using a UV/VIS spectrophotometer (Jenway, Keison Products, Chelmsford, UK) as described previously [41]. The anthocyanin content was determined using the formula *Q* = (A_530_ − 0.25A_657_) × *M*^−1^ (*Q*: anthocyanin yield; A_530_ and A_657_: absorption at the indicated wavelengths; *M*: mass of the plant). The leaves of each F_3_ plant were ground using liquid nitrogen, and 100 mg of the resulting powder was used for chlorophyll measurements. Chlorophyll content was determined following the method outlined by Hong et al. (2018) [41]. To determine the chlorophyll and carotenoid levels, samples of homogenized 14-day-old wheat seedlings were suspended in 100% acetone at 4 °C in the dark [42]. The homogenized samples were centrifuged at 12,000× *g* for 10 min, and the supernatant was used for pigment determination. The absorbance of the supernatant was recorded at 470, 644.8, and 661.6 nm using a UV/VIS spectrophotometer. The chlorophyll content was estimated using the extinction coefficients provided by Lichtenthaler (1987) [42].

### 4.3. Grain Color Determination

The color of wheat grains was determined using the L*, a*, and b* color scale with a ColorMate spectrophotometer (SCINCO, Seoul, Republic of Korea). Before the color measurement, the instrument was calibrated with standard black and white tiles. Each seed sample was placed in a Petri dish prior to reading the color parameters. The color L*, a*, and b* values were monitored and measured using embedded software (ColorMaster software 2017) in the device with three technical replicates.

### 4.4. Genotyping and SNP Calling

For the genotyping assay, leaves were sampled from each F_3_ population and stored at −80 °C until use. DNA was extracted from a single plant from each germplasm following the CTAB method outlined in the USDA instructor’s manual [43]. The extracted DNA was sent to the USDA-ARS Small Grain Genotyping Center in Fargo, ND (https://wheat.pw.usda.gov/GenotypingLabs/fargo; accessed on 7 March 2022) for processing using the Illumina iSelect 90K SNP Assay (Illumina, San Diego, CA, USA). SNP allele clustering and genotype calling were performed using GenomeStudio Module Polyploid Genotyping 2.0 software (https://support.illumina.com/downloads/genomestudio-2-0.html, accessed on 12 June 2023). Markers with minor allele frequencies <0.05 and missing data >10% were removed, resulting in 3969 high-quality SNPs for population structure and genome-wide association analyses. Following filtering, missing genotypes were imputed using BEAGLE v4.1 with the default settings [44].

### 4.5. Population Structure and LD

The program STRUCTURE v2.3.4, a model-based Bayesian cluster analysis tool was employed to infer the population structure [45]. The analysis involved 5000 burn-in periods followed by 50,000 Markov chain Monte Carlo iterations, ranging from 1 to 10 clusters (K), to identify the optimal K. Three independent runs were conducted for each K, and the most likely subgroups were determined by assessing the estimated likelihood values (ΔK) using Structure Harvester [46]. LD between marker loci on each chromosome was assessed with the squared allele frequency correlation (r^2^) using standalone TASSEL v.5.0 [47] and visualized using R. The LD decay distance was determined by fitting a non-linear model following the procedure described by Remington et al. [48], with an r^2^ threshold set at 0.1 and r^2^ equal to half of the maximum LD value.

### 4.6. GWASs

For GWASs of SNPs related to seed color, the GAPIT R package (version 3.0) was used [49]. Two GWAS methods were applied, namely, fixed and random model circulating probability unification (FarmCPU) and Bayesian-information and linkage-disequilibrium iteratively nested keyway (BLINK) [50,51]. In total, 3969 SNPs obtained after filtering were used for GWASs. To visualize the false positives of the implemented methods, Q–Q and Manhattan plots were generated using the internal program within GAPIT. The Manhattan plots depict the genomic distribution of marker associations, whereas the Q–Q plots assess the observed versus expected *p*-values. A stringent threshold of −log_10_P of 5.0 was applied to ensure robust identification of significant MTAs across the implemented genome-wide association study methods.

### 4.7. Transcriptome Data Analysis

Deep purple wheat and yellow wheat were cultivated in a radiation breeding research farm located at 35.5699° N and 126.9722° E (Jeongeup, Republic of Korea). The spikes were tagged at flowering time, and the grains were harvested at 10, 20, and 30 days after flowering. The samples were stored at −80 °C until further use. Total RNA was isolated from developing wheat seeds collected 10 days after flowering (DAF), 20 DAF, and 30 DAF using Meng and Feldman’s method [52]. An RNeasy plus micro kit (Qiagen, Hilden, Germany) was used to purify total RNA. Total RNA was isolated from developing wheat seeds collected 10 DAF, 20 DAF, and 30 DAF to construct RNA-Seq paired-end libraries using the TruSeq RNA sample preparation kit (Illumina). Each library was sequenced using the Illumina HiSeq2000 platform. The raw reads were preprocessed using Trimmomatic v0.36 to remove adapter sequences and low-quality bases [53]. The preprocessed reads were mapped to a high-quality wheat (*T. aestivum* L.) reference genome (International Wheat Genome Sequencing Consortium) from IWGSC using HISAT2 v2.1 [54,55]. The alignment was capable of determining alternative spliced transcripts for gene models based on IWGSC RefSeq v1.1. The HTSeq v0.6.1 high-throughput sequencing framework was employed to count the number of reads mapped to the exons of each gene [56]. DEGs were determined by *p* < 0.05, false discovery rate < 0.05, and absolute fold change >4 using edgeR [57] in the Bioconductor package. DEGs were identified by pairwise comparison at each time point between yellow and purple seeds. The log2-transformed transcript per million values was calculated using TPMCalculator and used to construct heatmaps of DEGs under yellow and purple wheat [58]. To identify genes associated with each agronomic trait, high-confidence annotated genes located within ±250 kb of each identified marker–trait association (MTA) were selected from the transcriptome data. The heatmap of gene expression was generated using MeV software, version 4.9.0 [59].

### 4.8. Gene Expression Analysis

RT-qPCR was performed using Bio-Rad CFX Opus 96 (Bio-Rad, Hercules, CA, USA) and TB Green premix EX Taq II (Takara, Tokyo, Japan). RT-qPCR primers for the indicated genes were designed using an oligonucleotide properties calculator. Each PCR reaction mixture (20 µL) contained 10 µL of 2 × TB Green premix, 1 µL of the first-strand cDNA, and gene-specific primers. The reactions were performed in the Bio-Rad CFX Opus 96 system under the following conditions: 30 s of denaturation at 95 °C, followed by 40 cycles of PCR amplification at 95 °C for 10 s and 65 °C for 30 s. The primers are presented in Table S1.

## 5. Conclusions

Overall, our comprehensive investigation into the genetic basis of seed color in wheat uncovered eight significant MTAs related to colorimetric traits (L*, a*, and b*) and candidate genes associated with seed coloration. The identified MTAs and candidate genes, including those encoding putative components of the MBW complex and E3 ubiquitin ligases, provide valuable insights into the molecular mechanisms governing seed color in wheat. Further investigations are essential to validate these correlations and unveil the precise roles of the identified genes in determining wheat seed color. This research provides a foundation for future studies to unravel the intricate molecular mechanisms governing the diverse colors of wheat seeds.

## Figures and Tables

**Table 1:** Marker–trait associations detected for L*, a*, and b*.

Trait	Associated SNP	Chromosome	Position	*p*-Value	MAF	Effect	PVE (%)
BLINK_a	BS00010988_51	2A	749,036,656	1.16 × 10^−7^	0.418831	6.525266	13.05690936
FarmCPU_L	BS00067992_51	2B	678,825,642	3.73 × 10^−8^	0.425325	−6.39214	52.66084028
BLINK_a	BS00067992_51	2B	678,825,642	4.64 × 10^−8^	0.425325	−2.73091	6.486490964
BLINK_a	D_contig30784_461	2A	464,937,628	1.16 × 10^−7^	0.418831	6.525266	4.701598626
BLINK_L	Ra_c13247_528	2A	678,678,921	2.58 × 10^−7^	0.483766	1.886488	19.93782288
BLINK_a	Ra_c13247_528	2A	678,678,921	1.12 × 10^−5^	0.483766	0.446553	0.178450817
FarmCPU_L	RAC875_c37638_233	4B	64,861,447	2.10 × 10^−6^	0.324675	2.037729	5.996921948
FarmCPU_a	RAC875_rep_c105150_1024	2A	758,487,818	7.45 × 10^−6^	0.422078	2.705539	86.07830835
BLINK_a	RAC875_rep_c105150_1024	2A	758,487,818	7.92 × 10^−13^	0.422078	3.01244	49.33097143
BLINK_a	RAC875_rep_c114597_51	2A	688,455,016	1.16 × 10^−7^	0.418831	−6.52527	14.33115573
BLINK_b	Tdurum_contig5114_319	2A	667,284,274	3.57 × 10^−7^	0.483766	1.242985	18.95993048

BLINK, Bayesian-information and linkage-disequilibrium iteratively nested keyway; FarmCPU, fixed and random model circulating probability unification; PVE, phenotype variance explained; SNP, single nucleotide polymorphism; MAF, minor allele frequency.

**Table 2:** Identification of genetic loci associated with phenotypic traits of wheat (*Triticum aestivum* L.) based on genome-wide association studies. BLINK, Bayesian-information and linkage-disequilibrium iteratively nested keyway; FarmCPU, fixed and random model circulating probability unification.

Trait	Marker	Gene ID	Chromosome	Annotation	Species	*p*-Value (BLASTP)
BLINK_a	BS00010988_51	TraesCS2A02G532800	2A	Diacylglycerol kinase 5-like	*Aegilops tauschii* subsp. *tauschii*	0
BLINK_L	Ra_c13247_528	TraesCS2A02G424200	2A	Unnamed protein product	*Triticum aestivum*	0
BLINK_L	Ra_c13247_528	TraesCS2A02G424100	2A	F-box protein At4g22660	*Aegilops tauschii* subsp. *tauschii*	6 × 10^−141^
BLINK_L	Ra_c13247_528	TraesCS2A02G424600	2A	Cinnamyl alcohol dehydrogenase 7-like	*Aegilops tauschii* subsp. *tauschii*	0
FarmCPU_L	RAC875_c37638_233	TraesCS4B02G070600	4B	Predicted protein	*Hordeum vulgare* subsp. *vulgare*	0
FarmCPU_L	RAC875_c37638_233	TraesCS4B02G070800	4B	E3 ubiquitin-protein ligase RING type	*Aegilops tauschii* subsp. *tauschii*	0
FarmCPU_L	RAC875_c37638_233	TraesCS4B02G071000	4B	Cinnamoyl-CoA reductase	*Aegilops tauschii* subsp. *tauschii*	5.00 × 10^−179^
FarmCPU_a	RAC875_rep_c105150_1024	TraesCS2A02G551200	2A	Uracil DNA glycosylase, mitochondrial	*Aegilops tauschii* subsp. *tauschii*	0
FarmCPU_a	RAC875_rep_c105150_1024	TraesCS2A02G551900	2A	Ubiquinone biosynthesis protein COQ4 homolog	*Aegilops tauschii* subsp. *tauschii*	9 × 10^−168^
FarmCPU_a	RAC875_rep_c105150_1024	TraesCS2A02G551700	2A	F-box protein At5g65850	*Aegilops tauschii* subsp. *tauschii*	5 × 10^−93^
FarmCPU_a	RAC875_rep_c105150_1024	TraesCS2A02G552400	2A	MYB transcription factor 77	*Aegilops tauschii* subsp. *tauschii*	0
BLINK_a	RAC875_rep_c114597_51	TraesCS2A02G436300	2A	Leucine-rich repeat extensin-like protein 3	*Brachypodium distachyon*	7 × 10^−77^
BLINK_a	RAC875_rep_c114597_51	TraesCS2A02G436800	2A	Serine/threonine protein kinase minibrain-like	*Aegilops tauschii* subsp. *tauschii*	0
BLINK_a	RAC875_rep_c114597_51	TraesCS2A02G436200	2A	Pectin acetylesterase 7	*Aegilops tauschii* subsp. *tauschii*	0
BLINK_a	RAC875_rep_c114597_51	TraesCS2A02G435800	2A	FBD-associated F-box protein At5g50270	*Aegilops tauschii* subsp. *tauschii*	0
BLINK_b	Tdurum_contig5114_319	TraesCS2A02G409400	2A	bHLH transcription factor	*Triticum aestivum*	0
BLINK_b	Tdurum_contig5114_319	TraesCS2A02G409600	2A	Anthocyanin regulatory R-S protein (MYC protein)	*Triticum urartu*	0

## Data Availability

The dataset is available upon request from the authors.

## Supplementary Materials

The supporting information can be downloaded at: https://www.mdpi.com/article/10.3390/ijms25073600/s1.
