# Supplementary material for: Genome-Wide Association Study to Identify Marker–Trait Associations for Seed Color in Colored Wheat (Triticum aestivum L.)
Source: Int J Mol Sci. 2024 Mar 22;25(7):3600. doi: 10.3390/ijms25073600 (PMC11011601; doi:10.3390/ijms25073600)
Supplement: Supplementary file 1 [file ijms-25-03600-s001.zip › Figure S1.pdf]

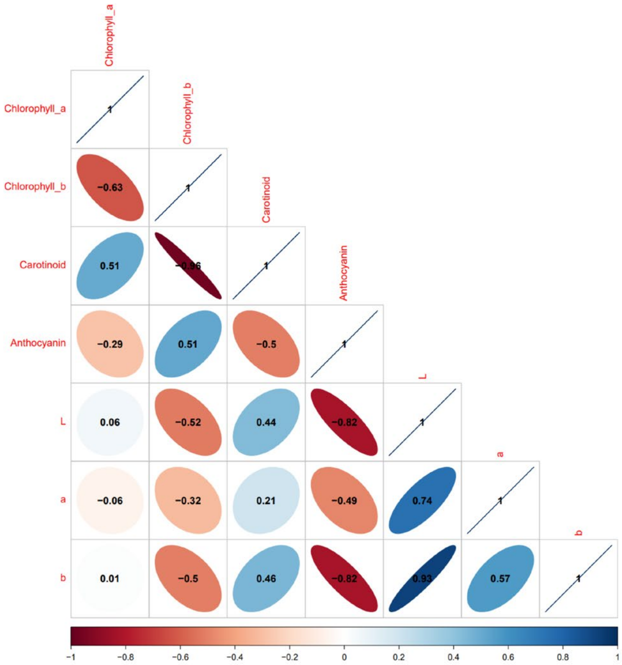

Figure S1. Pearson's correlation coefficients among the phenotypic traits are depicted, highlighting their interrelationships.
